# Comprehensive analysis of GPN1 in human cancer and its effects on the migration of hepatocellular carcinoma cells

**DOI:** 10.17305/bb.2024.11310

**Published:** 2024-11-08

**Authors:** Rongtao Zhu, Senfeng Zhao, Jiahui Cao, Yin Liu, Ruopeng Liang

**Affiliations:** 1 Department of Hepatobiliary and Pancreatic Surgery, The First Affiliated Hospital of Zhengzhou University, Zhengzhou, China

**Keywords:** GPN1, hepatocellular carcinoma, prognosis

## Abstract

GPN1 has emerged as a potential key player in cancer biology, influencing tumor progression, prognosis, and response to immunotherapy. To investigate the prognostic value of GPN1 in cancer and its role in the migration of hepatocellular carcinoma (HCC or LIHC) cells, we used several databases to assess GPN1 expression levels and effects in human tumors. Furthermore, experiments were conducted to verify changes in GPN1 expression in HCC cell lines and explore its biological function. We found that the GPN1 gene and protein expression were significantly increased in several tumor tissues. Higher GPN1 expression was associated with unfavorable overall survival (OS). Additionally, there was a strong association between GPN1 expression and several clinicopathological features, according to multivariate Cox regression analysis. Moreover, GPN1 gene mutation and methylation were present in some tumors. A relationship was also found between GPN1 expression and immune infiltration. Notably, immune checkpoint analysis showed that GPN1 expression was correlated with PD-1/PDL-1 and cytotoxic T-lymphocyte-associated protein 4 (CTLA-4), suggesting it may serve as a biomarker for predicting immune subtypes and response to immunotherapy in HCC. Enrichment analysis in HCC indicated that GPN1 is primarily involved in RNA metabolism. Additionally, drug sensitivity analysis revealed that GPN1 appeared to be responsive to 16 drugs. Finally, GPN1 upregulation was confirmed to promote the migration of HCC cells. This study provides a comprehensive overview of GPN1 in human cancer and demonstrates that GPN1 contributes to the migration of HCC cells, potentially serving as a prognostic and immunotherapy biomarker.

## Introduction

Cancer is one of the leading causes of mortality worldwide, presenting a major obstacle to increasing life expectancy [1]. In 2020, liver cancer was a primary contributor to cancer-related deaths, accounting for approximately 8.3% of total cases. Hepatocellular carcinomas (HCCs), the most common type of primary liver cancer, make up 75%–85% of cases [2]. Despite advancements in treatment, the prognosis for HCC remains poor, particularly for patients with advanced-stage disease [3]. New drugs have led to progress in targeted therapies and immunotherapy for HCC. Additionally, a systematic treatment model combining transarterial chemoembolization (TACE) with other therapies, such as immunotherapy, has been applied to patients with HCC [4]. However, only a small percentage of patients experience moderate benefits from this multidisciplinary approach [3, 5, 6]. Identifying biomarkers that can predict prognosis and treatment response is therefore crucial for effective HCC subtyping, as is further investigation into the underlying pathogenic mechanisms.

The synthesis of mRNA and small nuclear RNA (snRNA) by RNA polymerase II (RNAP II) is essential for maintaining normal cellular function. GTPases interact directly with several RNAP II subunits, including Rpb4, Rpb7, Rpb2, and Rpb1 [7, 8]. These GTPases, as stable interaction partners of RNAP II, play a key role in the biogenesis and nuclear transport of RNAP II, as well as its nuclear targeting [8]. The GPN-loop GTPase (GPN) family, which includes GPN1, GPN2, and GPN3, is particularly important in this context. GPN1 (also known by aliases, such as XAB1, MBDIN, NTPBP, RPAP4, and ATPBD1A) has a highly conserved loop unique to GPN proteins [9–12] and is involved in maintaining RNAP II stability and function, as well as nucleotide excision repair [7, 13]. In *Saccharomyces cerevisiae*, the carboxy-terminal tail of GPN1 is critical for microtubule stability and function [14]. While GPN3 mutations are known to cause significant functional changes in various cancers [15], the expression, prognostic relevance, and biological function of GPN1 in human cancers—particularly in HCC—remain unclear.

Identification of gene–tumor interactions has been greatly advanced by genomic technologies. Using publicly available data, we found that GPN1 expression differed significantly between various tumor types and normal tissues. A multi-bioinformatics approach was used to examine the diagnostic and prognostic value of GPN1, as well as its clinicopathological features, particularly in HCC. The genetic mutations and the relationship between GPN1 expression and immune cell infiltration in tumors were then assessed, including the impact of immune subtypes and therapeutic responses in HCC. Additionally, analyses of the protein–protein interaction (PPI) network and molecular enrichment highlighted roles for GPN1-related molecules, offering insights for future research on their mechanisms. Furthermore, the relationship between GPN1 expression and drug sensitivity was analyzed. Finally, GPN1 expression was evaluated, and its biological function in HCC cells was confirmed. In summary, our pan-cancer analysis, conducted through bioinformatics, explored the diagnostic and prognostic significance of GPN1 and demonstrated its role in HCC cell migration.

## Materials and methods

### GPN1 gene and protein expression analysis

GPN1 gene and protein expression analysis was conducted in human tumors and normal tissues using the Tumor Immune Estimation Resource 2.0 (TIMER2.0) database (http://timer.cistrome.org/) [16]. Additionally, data from The Cancer Genome Atlas (TCGA) and the Genotype-Tissue Expression (GTEx) database were included in this study. Analysis and visualization of paired tumor/normal samples were performed using R (version 4.0.4) and the ggplot2 package. Protein expression analysis was also carried out on several samples from the Clinical Proteomic Tumor Analysis Consortium (CPTAC) database (https://cptac-data-portal.georgetown.edu/cptacPublic/) [17]. To assess GPN1 protein expression, we used data from the Human Protein Atlas (HPA) (https://www.proteinatlas.org/). GPN1 protein expression was identified in tissues from three patients with HCC and three patients with renal cell carcinoma. These tissue samples were surgically resected and pathologically confirmed.

### Survival analyses of GPN1

The correlation between GPN1 expression and overall survival (OS) was assessed using Kaplan–Meier plots (https://kmplot.com/analysis/) [18]. To evaluate the prognostic value of GPN1 expression across various datasets, we utilized the PrognoScan database (http://dna00.bio.kyutech.ac.jp/PrognoScan/). Receiver operating characteristic (ROC) analysis and area under the curve (AUC) calculations were performed with the R package pROC to determine the diagnostic and prognostic value of GPN1 in human cancers. To further explore the prognostic significance of GPN1 in liver cancer specifically, Kaplan–Meier survival analysis was conducted using data from the Gene Expression Omnibus (GEO) and TCGA databases (datasets GSE76427, GSE54236, TCGA-LIHC). Additionally, time-dependent ROC analysis was employed to evaluate the prognostic impact of GPN1 on HCC.

### Expression of GPN1 and clinicopathological characteristics in HCC

TCGA-HCC data were used to extract clinicopathological information about patients with HCC. Key parameters—including OS, T stage, alpha-fetoprotein (AFP) levels, histological grade, and pathological stage—were analyzed using R package. Expression levels of GPN1 were assessed in relation to these clinicopathological characteristics through univariate and multivariate Cox regression analyses. Statistical visualizations were generated with the ggplot2 package in R.

### GPN1 genetic alteration and DNA methylation in cancer

Using the cBioPortal database (https://www.cbioportal.org/) [19], we assessed the alteration frequency of GPN1 in TCGA cancer samples. Specifically, we analyzed TCGA tumor samples for mutation types, mutation sites, and three-dimensional structural changes. Using R and ggplot2, we examined the correlation between GPN1 genetic alterations (single nucleotide variations [SNVs] and copy number variations [CNVs]) and their impact on HCC. DNA methylation of GPN1 in HCC was evaluated through the UALCAN data analysis portal (http://ualcan.path.uab.edu/analysis.html). We also assessed the prognostic role of GPN1 DNA methylation status in HCC using the gene set co-expression analysis (GSCA) database (http://bioinfo.life.hust.edu.cn/GSCA/#/) [20].

### Expression of GPN1 and immune infiltration

Gene Set Variation Analysis (GSVA) was performed as a single-sample Gene Set Enrichment Analysis (GSEA) to investigate correlations between GPN1 expression and immune cell presence across various TCGA cancers. To assess immune cell infiltration related to GPN1, we utilized the TIMER database (cistrome.shinyapps.io/timer). Correlations between GPN1 and immune checkpoints were visualized using the R packages corrplot and ggplot2. Additionally, the TISIDB database (http://cis.hku.hk/TISIDB/index.php) was employed to examine the association between GPN1 expression and HCC subtypes characterized by immune and molecular features [21]. This analysis identified several immune subtypes: C1 (wound healing), C2 (interferon-gamma dominant), C3 (inflammatory), C4 (depleted lymphocytes), C5 (immunologically quiet), and C6 (dominant transforming growth factor beta), along with three molecular subtypes. Furthermore, we assessed the potential of GPN1 expression to guide immunotherapy response in HCC using data from the Riaz cohort (2018) and IMvigor cohort (2018), accessible via R packages.

### Functional enrichment analysis of GPN1 in HCC

The top 100 molecules interacting with GPN1 across various cancer types were identified using the Gene Expression Profiling Interactive Analysis (GEPIA) database (http://gepia2.cancer-pku.cn/#index). PPI networks involving GPN1 were constructed and visualized using Cytoscape (v3.9.0) [22, 23]. Functional enrichment analyses were conducted to elucidate the molecular mechanisms of GPN1 in HCC. Gene Ontology (GO) and Kyoto Encyclopedia of Genes and Genomes (KEGG) enrichment analyses were used to identify the biological functions and pathways associated with GPN1 in HCC. Additionally, the GSEA of GPN1 in HCC was performed using the MSigDB ClusterProfiler package (version 4.0.4).

### GPN1 expression and drug sensitivity

The half-maximal inhibitory concentration (IC50) of various drugs was calculated using the pRRophetic R package to analyze the relationship between drug response and GPN1 expression in HCC. Additionally, 96 drugs—including both targeted therapies and small-molecule compounds—were evaluated using the ggplot2 R package to assess their effects.

### Plasmid construction and transfection

The knockdown of GPN1 using shRNA was achieved through OBiO Technology (Shanghai, China) with the pCLenti-U6-shRNA-CMV-EGFP-WPRE vector. The target sequences for human GPN1 mRNA were as follows: sh-NC: TTCTCCGAACGTGTCACGT, sh-GPN1-1: CCTTGAATCAAGAGACTACAT, and sh-GPN1-2: GCCCAGAACATGTCCAAATAT. Transfection of the plasmid into HCC cells was performed using Lipofectamine 2000 (Thermo Fisher Scientific). After 72 h, proteins were extracted, and Western blotting (WB) was conducted to evaluate the transfection efficiency.

### Preliminary experiment and functional verification of GPN1 in HCC cells

Three HCC cell lines (Huh-7, SMMC7721, and HCCLM3) and one normal human hepatocyte cell line (THLE-2) were obtained and cultured in Dulbecco’s Modified Eagle Medium containing 10% fetal bovine serum. Total RNA was extracted from THLE-2 cells and HCC cell lines, followed by quantitative PCR (qPCR) with the SYBR Green PCR Kit. Next, cells were collected to extract protein, followed by western blot analysis of GPN1. The BCA Protein assay kit was used for protein concentration evaluation. To verify the role of GPN1 in the migration of HCC cells, a wound healing assay and transwell assay were conducted. Short hairpin-GPN1-1 (Sh-GPN1-1), sh-GPN1-2 and sh-NC were transfected in SMMC7721 and HCCLM3 cells, respectively. The cells transfected with different plasmids were used for wound healing assay and transwell after transfection for 72 h. For the wound healing assay, the pipette tip was employed to draw the surface of the cell layer. The distance of the injury area at 24 h was measured. The relative migration rate was calculated by normalizing the distance of the injury area at 0 h. Migration assays were conducted in transwell chambers. Cells were injected into the upper layer, which were cultured in suitable conditions for 48 h. We counted the cells on the bottom of the chambers.

### Ethical statement

This study was performed with the approval of the Ethics Committee of the First Affiliated Hospital of Zhengzhou University (2022-KY-1519).

### Statistical analysis

Several bioinformatics databases and R version 4.0.4 were used for bioinformatics analyses. To assess the prognostic value of GPN1, we applied Fisher’s exact test, Chi-square test, Spearman’s test, and univariate and multivariate Cox analyses to examine the correlation between GPN1 expression and clinicopathologic characteristics. *P* < 0.05 was considered statistically significant.

## Results

### Expression of GPN1 in pan-cancer

**Figure 1.**
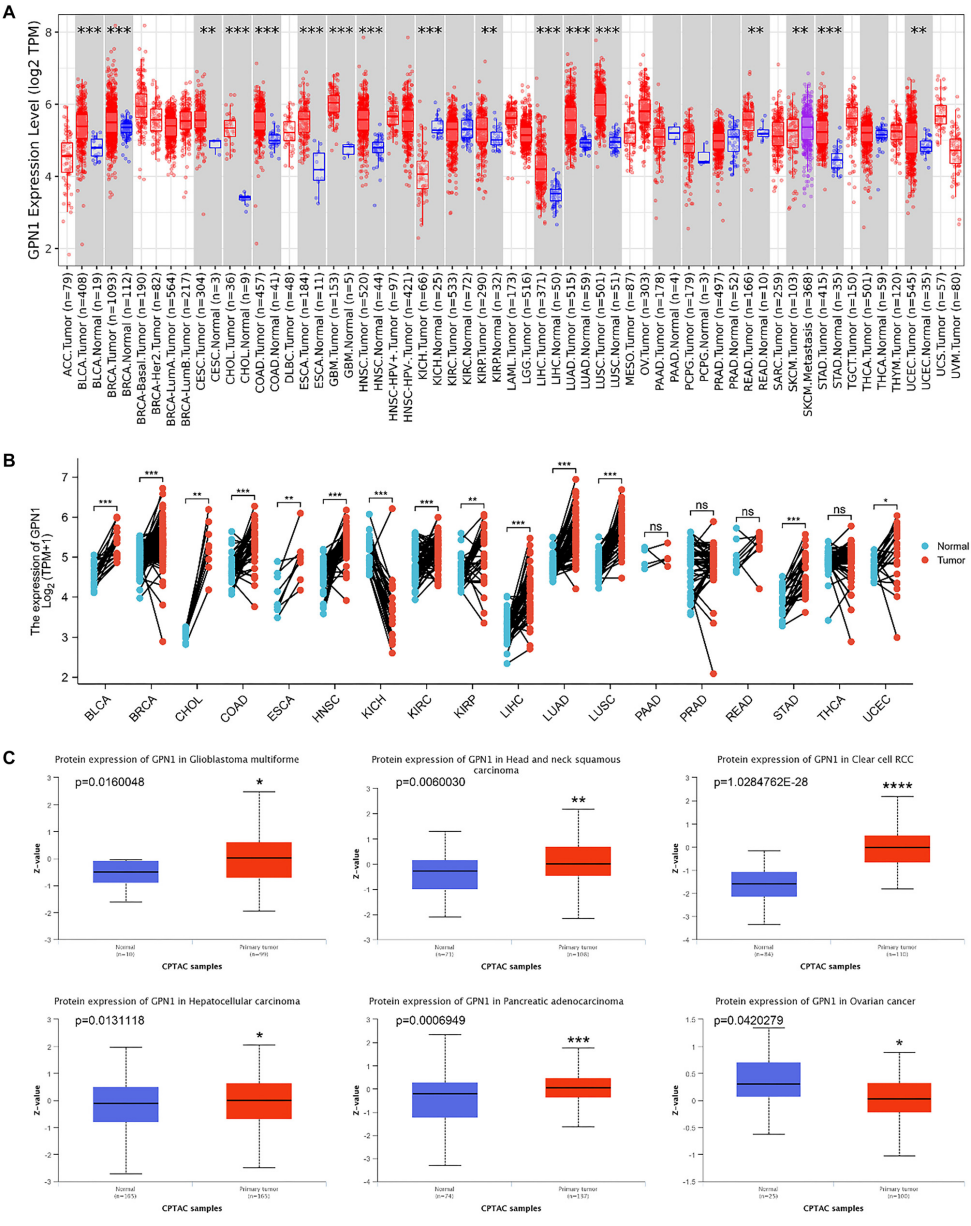
**GPN1 gene and protein expression in pan-cancer.** (A) Expression of GPN1 in pan-cancer in the TIMER database; (B) Expression of GPN1 in pan-cancer paired tumor/normal samples in TCGA-GTEx; (C) Protein expression of GPN1 in the CPTAC database; (D) Protein expression of GPN1 in tumor and normal tissues in the HPA database. TCGA: The cancer genome atlas; GTEx: Genotype-tissue expression; CPTAC: Clinical proteomic tumor analysis consortium; HPA: Human protein atlas; TIMER: Tumor immune estimation resource.

The expression of GPN1 in 28 cancers in the TIMER2.0 database was analyzed. Nineteen tumor types showed a significant increase in GPN1 expression compared to the control group, while only kidney chromophobe (KICH) displayed lower GPN1 expression (Figure 1A). As a control, we validated this gene expression pattern in normal tissue using the GTEx dataset. Consistent with the TIMER2.0 findings, GPN1 expression was elevated in 13 cancer types but reduced in KICH (Figure 1B).

To examine GPN1 protein expression, we used data from the CPTAC database. GPN1 protein levels were significantly different across several cancers, including glioblastoma (GBM), head and neck squamous cell carcinoma (HNSC), kidney renal clear cell carcinoma (KIRC), HCC, ovarian serous cystadenocarcinoma (OV), and pancreatic adenocarcinoma (PAAD). Interestingly, OV exhibited lower protein expression of GPN1 compared to normal samples (Figure 1C).

### Protein expression of GPN1 in liver and renal tumors

**Figure 2.**
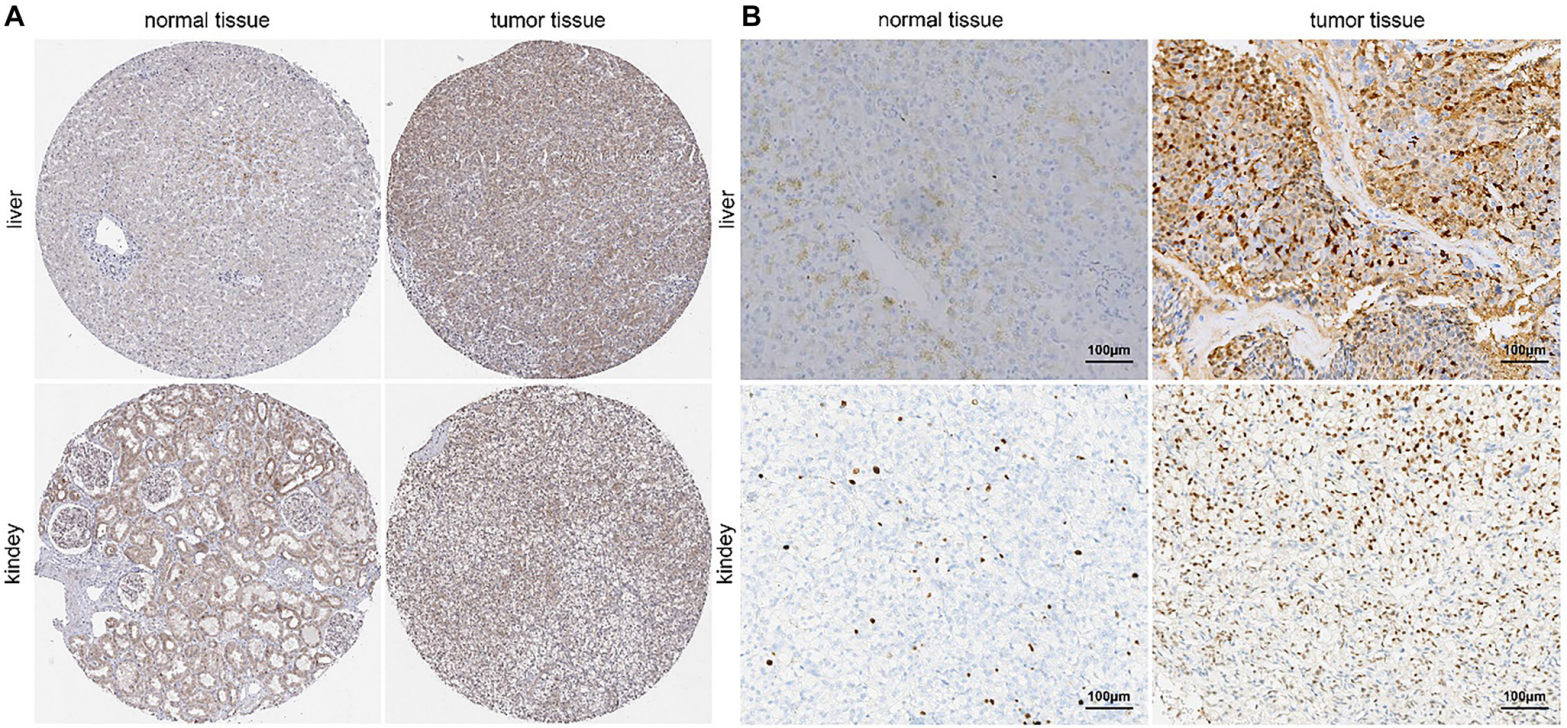
**Protein expression of GPN1 in liver and renal tumors.** (A) GPN1 expression levels in kidney/ KIRP and liver/HCC based on the HPA database; (B) Protein expression of GPN1 in patients with HCC and KIRP. HCC: Hepatocellular carcinoma; HPA: Human protein atlas.

Based on data from the HPA database, GPN1 expression levels differ between normal and tumor tissues, specifically in kidney (renal papillary cell carcinoma, KIRP) and liver (HCC) tissues (Figure 2A). Immunohistochemical (IHC) staining was conducted on tumor tissues and paired normal tissues to validate the differential expression of GPN1 at the protein level. As shown in Figure 2B, GPN1 protein was primarily localized in the cytoplasm, with some expression observed in the nucleus. We also found that GPN1 protein expression was significantly lower in paired normal tissues compared to liver and renal tumors. These findings confirm that GPN1 protein levels are upregulated in HCC and KIRP.

### Higher expression of GPN1 is associated with a poor prognosis

**Figure 3.**
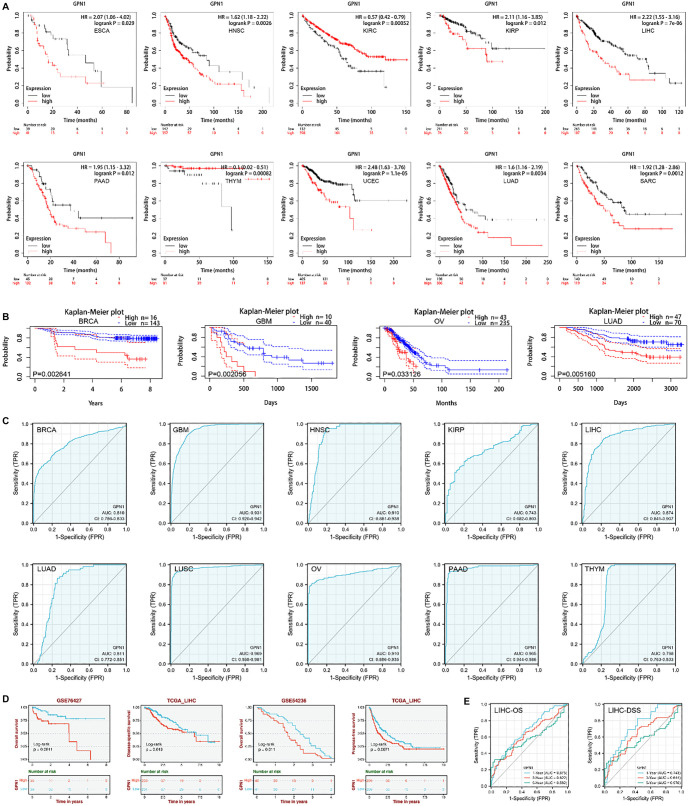
**Higher expression of GPN1 was correlated with a poor prognosis.** (A) High GPN1 expression had a poorer prognosis in Kaplan–Meier analyses; (B) High GPN1 expression had a poorer prognosis in PrognoScan databases. The solid lines represent the survival curve and the broken lines represent the 95% confidence interval; (C) The diagnostic value of GPN1 was evaluated using ROC curves in a variety of cancer types in TCGA database; (D) The correlation between GPN1 expression and prognosis (OS, DSS, and PFS) according to different datasets; (E) Time-dependent ROC curve verified the predictive effect of GPN1 on HCC. OS: Overall survival; DSS: Disease-specific survival; PFS: Progression-free survival; HCC: Hepatocellular carcinoma; TCGA: The cancer genome atlas; ROC: Receiver operating characteristic.

To evaluate the prognostic value of GPN1 in cancer, Kaplan–Meier analyses were performed. The results showed that in esophageal carcinoma, HNSC, KIRC, KIRP, HCC, lung adenocarcinoma (LUAD), PAAD, thymic carcinoma, sarcoma, and uterine corpus endometrial carcinoma (UCEC), higher expression of GPN1 was associated with poor prognosis (Figure 3A). Meanwhile, the PrognoScan database revealed that patients with GBM, LUAD, breast cancer, and OV who had high levels of GPN1 expression also had a poorer prognosis (Figure 3B). The diagnostic value of GPN1 was evaluated using ROC curves across a variety of cancer types. GPN1 showed good diagnostic accuracy, especially for GBM, HNSC, LUSC, OV, and PAAD (Figure 3C). To further assess the prognostic significance of GPN1 in liver cancer, we analyzed the correlation between GPN1 expression and prognosis, including OS, disease-specific survival (DSS), and progression-free survival (PFS), using different datasets. The GEO datasets (GSE76467 and GSE54236) showed that high GPN1 expression was associated with poor prognosis in HCC. Additionally, TCGA data (TCGA-LIHC) confirmed that higher GPN1 expression in HCC was linked to poor prognosis (DSS, PFS) (Figure 3D). A time-dependent ROC curve validated the predictive effect of GPN1 on HCC (Figure 3E). As shown in Figure 3E, the AUC values for predicted survival rates at one, three, and five years were moderate, with the predictive value gradually decreasing over time. In summary, these results demonstrate that GPN1 is valuable for predicting HCC prognosis, and high GPN1 expression may indicate a poor prognosis in HCC patients.

### GPN1 gene expression is associated with HCC clinical characteristics

**Figure 4.**
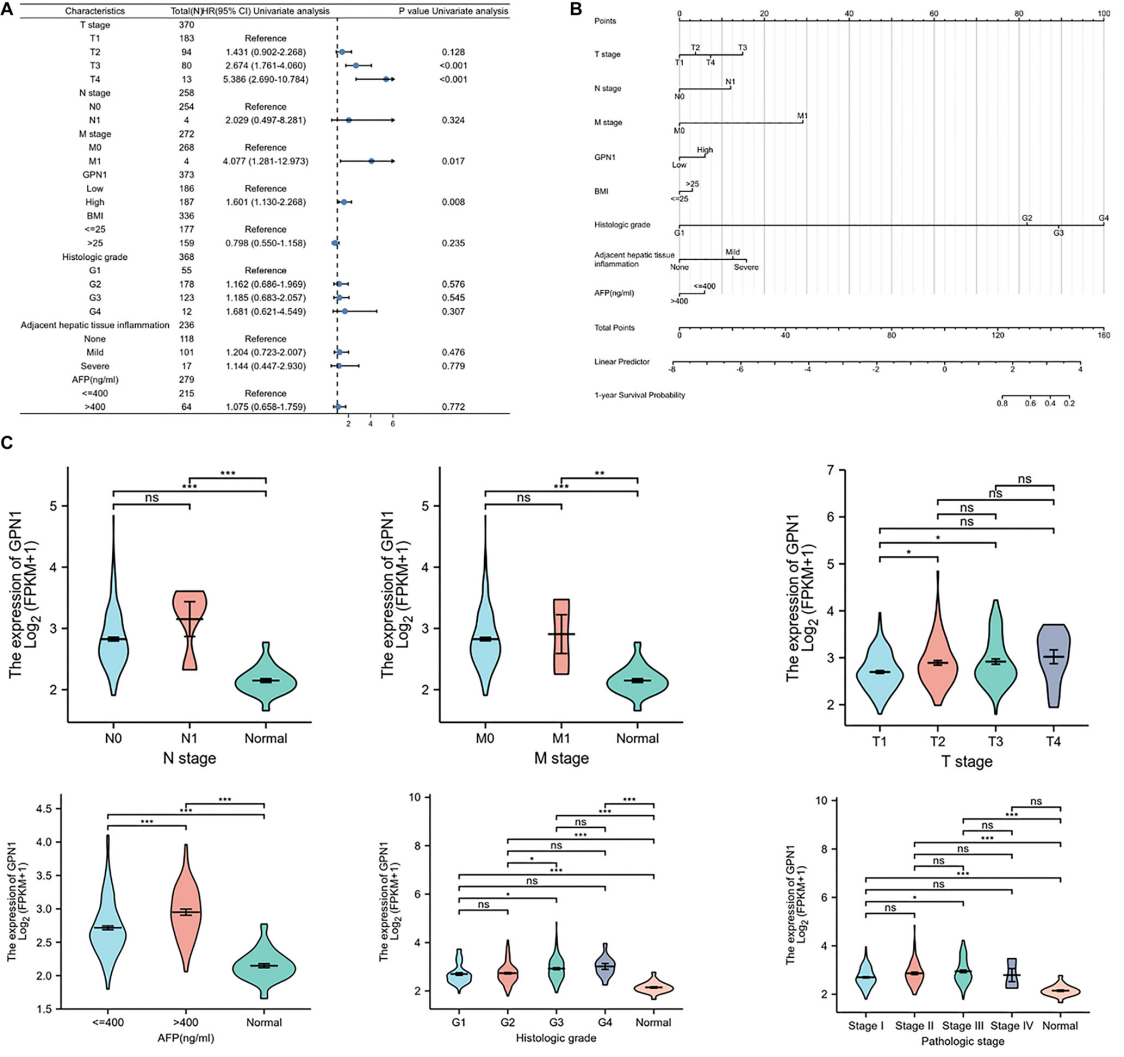
**GPN1 gene expression was associated with HCC clinical characteristics.** (A) Multivariate Cox regression analysis of prognosis-related risk factors in HCC; (B) Construction of a nomogram model to predict HCC progression; (C) Subgroup analysis of GPN1 expression in HCC. HCC: Hepatocellular carcinoma.

Clinical data from a total of 374 HCC samples were obtained from TCGA, and these samples were analyzed in subgroups based on various clinical characteristics. A significant association was found between GPN1 expression and histopathological T stage, histologic grade, and AFP levels in HCC (Table 1). Multivariate cox regression analysis of prognosis-related risk factors in HCC showed that OS was associated with T3 and T4 stages, M1 stage, and high GPN1 expression (Figure 4A).

**Table 1:** Association of GPN1 expression and the clinical characteristics of HCC

**Characteristics**	**Total (N)**	**OR (95% CI)**	***P* value**
Pathologic T stage (T2 & T3 & T4 vs T1)	371	2.030 (1.342–3.069)	**<0.001**
Pathologic N stage (N1 vs N0)	258	2.687 (0.276–26.173)	0.395
Pathologic M stage (M1 vs M0)	272	0.928 (0.129–6.685)	0.941
BMI (> 25 vs <═ 25)	337	0.687 (0.447–1.055)	0.087
Histologic grade (G4 & G3 vs G1 & G2)	369	2.148 (1.394--3.310)	**<0.001**
Adjacent hepatic tissue inflammation (mild & severe vs none)	237	1.128 (0.677–1.879)	0.645
AFP (ng/mL) (> 400 vs <═ 400)	280	3.185 (1.761--5.761)	**<0.001**
Fibrosis ishak score (1/2 & 3/4 & 5 & 6 vs 0)	215	1.338 (0.757–2.363)	0.316

AFP: Alpha-fetoprotein; BMI: Body mass index; GPN1: GPN-loop GTPase 1; HCC: Hepatocellular carcinoma.

To predict cancer prognosis, a nomogram model was constructed (Figure 4B), incorporating GPN1 expression levels, TNM stage, BMI, histologic grade, AFP, and adjacent hepatic tissue inflammation to assess the predictive contribution of each factor for HCC prognosis. Each factor was assigned a point value, and the total score was calculated to predict the 1-year survival probability for HCC patients. Furthermore, using normal samples from GTEx as a reference, subgroup analysis revealed a significant difference in GPN1 expression between HCC patients and controls, particularly notable in subgroups defined by histologic grade and AFP levels (Figure 4C). Although no statistically significant difference was observed within TNM stage subgroups, GPN1 expression was consistently higher in tumor tissues compared to normal tissues. Additionally, GPN1 expression showed significant variation among HCC patients with different AFP levels. Collectively, these findings indicate that GPN1 is associated with key clinical characteristics in HCC patients, and high GPN1 expression may be indicative of a poor prognosis for these patients.

### GPN1 gene alterations and DNA methylation

**Figure 5.**
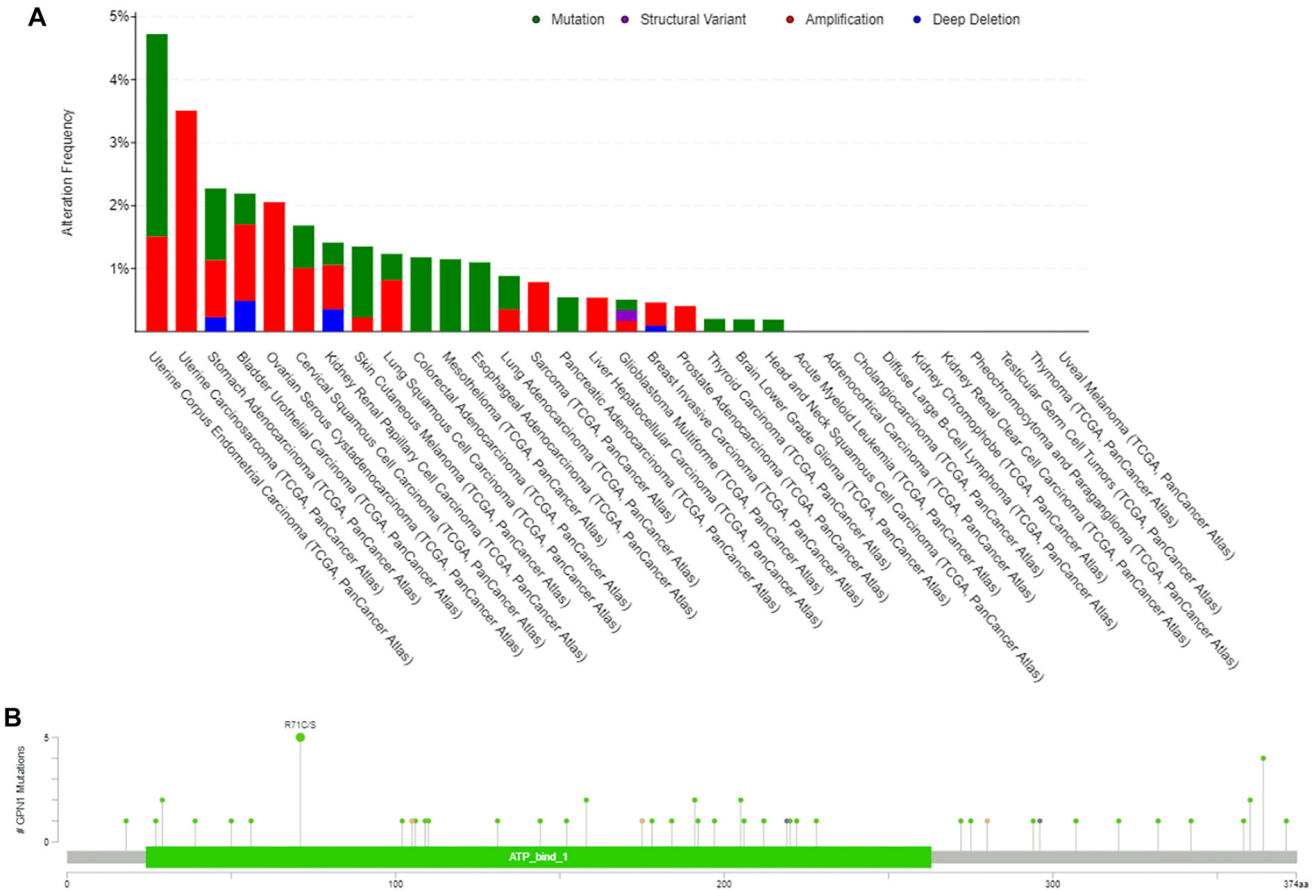
**GPN1 gene alteration in cancer.** (A) The alteration type and frequency of GPN1 in the cBioPortal database, including the structural variant data, mutation data, and CAN data; (B) The alteration site and frequency in GPN1.

According to the cBioPortal database, the GPN1 gene shows alterations across various cancers. A pan-cancer analysis indicated that the primary types of gene alterations were mutations and amplifications, with amplifications being particularly common in HCC samples (Figure 5A). The most frequent mutation site was the R7/CS alteration, observed in four UCEC cases and one colorectal cancer case (Figure 5B).

**Figure 6.**
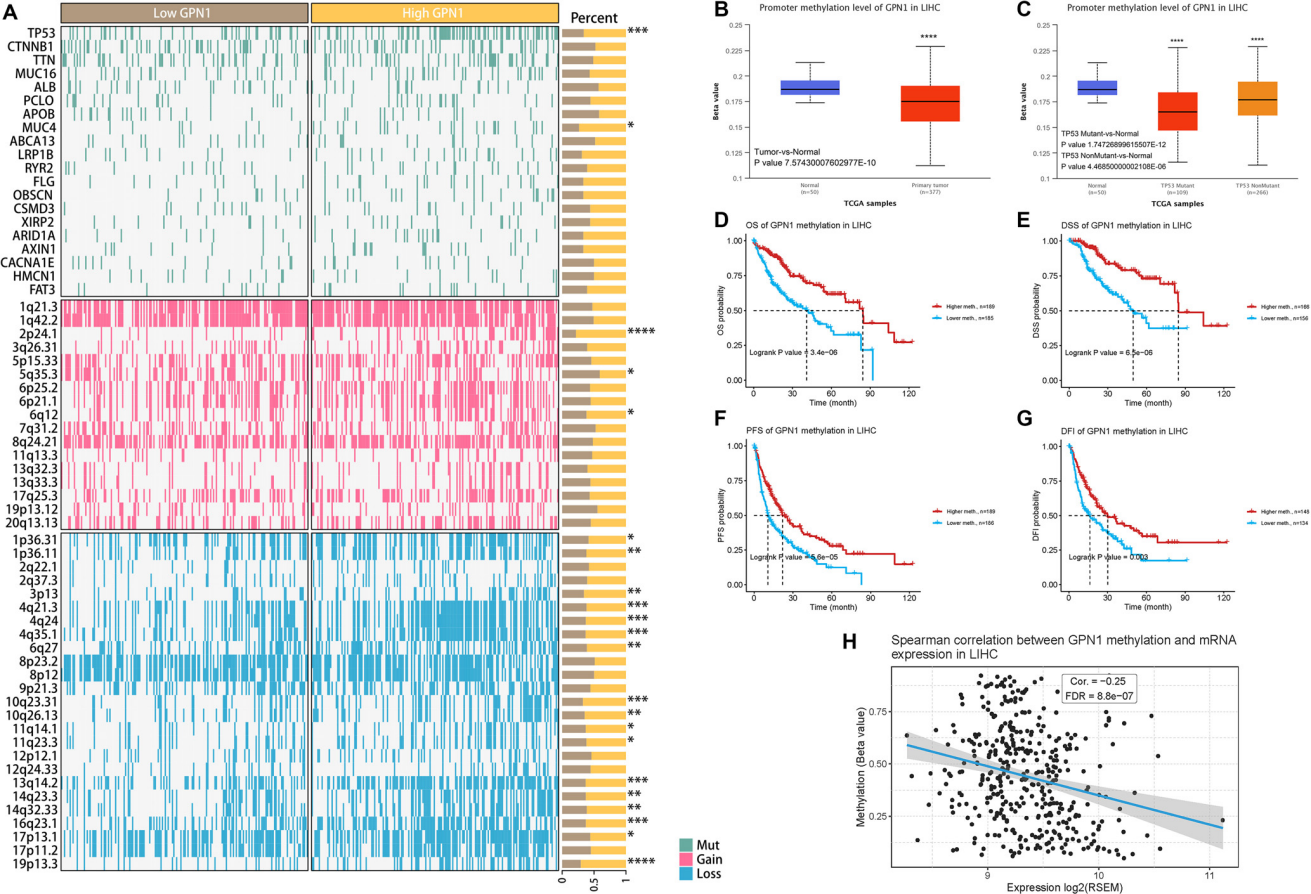
**GPN1 expression with SNVs and CNVs in HCC and DNA methylation of GPN1.** (A) The correlation of GPN1 expression with SNVs and CNVs in HCC; (B) GPN1 DNA methylation was reduced in HCC; (C) Methylation of GPN1 was correlated with TP53 mutations; (D–G) The prognosis of HCC was influenced by GPN1 DNA methylation in the GSCA database; (H) The coefficient of probes with cg15744128 for GPN1 in HCC. HCC: Hepatocellular carcinoma; GSCA: Gene set co-expression analysis; SNVs: Single nucleotide variations; CNVs: Copy number variations.

Using the TCGA database, we examined the correlation between gene SNVs and CNVs and GPN1 expression in HCC. We found that specific gene mutations, including those in tumor protein p53 (TP53) and mucin 4 (MUC4), as well as in loci on chromosomes 2p24.1, 4q21.3, 4q35.1, 13q14.2, and 19p13.3, were associated with variations in GPN1 expression in HCC (Figure 6A). Analysis using the UALCAN database revealed that GPN1 DNA methylation levels were significantly reduced in HCC and were strongly correlated with TP53 mutations (Figure 6B and 6C).

Furthermore, data from the GSCA database indicated that GPN1 DNA methylation significantly affects HCC prognosis (Figure 6D–6G). Notably, the correlation coefficient for probe cg15744128 was −0.250 in HCC (Figure 6H). These findings suggest that alterations in the GPN1 gene and its DNA methylation status may play important roles in genetic changes associated with HCC progression.

### Immune infiltration in HCC is correlated with GPN1

**Figure 7.**
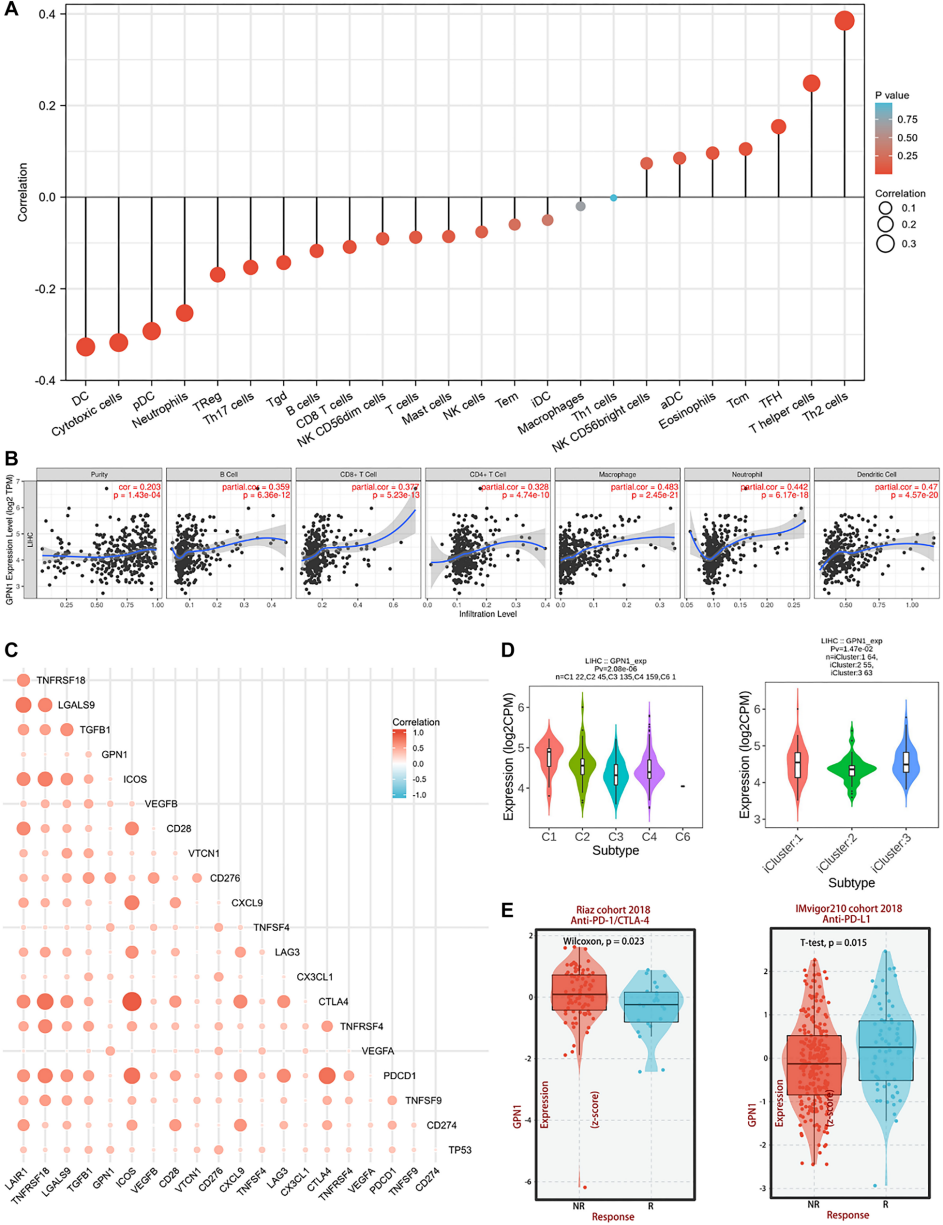
**Correlation between GPN1 and immune infiltration in HCC.** (A) Correlation between GPN1 expression and immune infiltration in HCC; (B) Analysis between GPN1 expression and immune cells in HCC in the TIMER database; (C) Correlation analysis of immune checkpoints with GPN1; (D) Correlation between GPN1 expression and molecular or immune subtypes in the HCC TISIDB database; (E) Expression of GPN1 was associated with the immunotherapy response with anti-PD-1/CTLA-4 or anti-PD-L1. HCC: Hepatocellular carcinoma; TIMER: Tumor immune estimation resource.

Using the TCGA database, we demonstrated a positive correlation between GPN1 expression and immune cell infiltration in HCC. Analysis and visualization were conducted using the R package GSVA, with results shown in Figure 7A. We found that higher GPN1 expression levels were positively associated with Helper T cells, particularly type 2 helper T cells (Th2). In contrast, GPN1 expression was negatively associated with dendritic cells (DCs), cytotoxic cells, plasmacytoid DCs (pDCs), and neutrophils.

To further investigate, we used the TIMER2.0 database to analyze the relationship between GPN1 expression and various immune cell types, finding correlations with CD8+ T cells, CD4+ T cells, and macrophages (Figure 7B). We also examined the correlation of GPN1 with 21 immune checkpoint genes. Notably, GPN1 expression showed a positive correlation with a cluster of differentiation 276 (CD276), programmed death-ligand 1 (PD-L1), TP53, tumor necrosis factor superfamily member 4 (TNFSF4), and vascular endothelial growth factor A (VEGFA) (Figure 7C).

Using the TISIDB database, we explored the association between GPN1 expression and both molecular and immune subtypes of HCC. Our results revealed differential GPN1 expression across HCC molecular subtypes, with significantly lower expression in the iCluster2 molecular subtype compared to others. Additionally, we observed that various immune subtypes were associated with GPN1 expression levels (Figure 7D).

Finally, based on data from the Riaz cohort (2018) and the IMvigor210 cohort (2018), we found that differential GPN1 expression was linked to responses to immunotherapies targeting programmed cell death protein 1 (PD-1)/cytotoxic T-lymphocyte-associated protein 4 (CTLA-4) and PD-L1, respectively (Figure 7E). These findings suggest that GPN1 expression may serve as a useful biomarker for selecting immunotherapy options in HCC.

### Functional enrichment analysis and pathways of GPN1 in HCC

**Figure 8.**
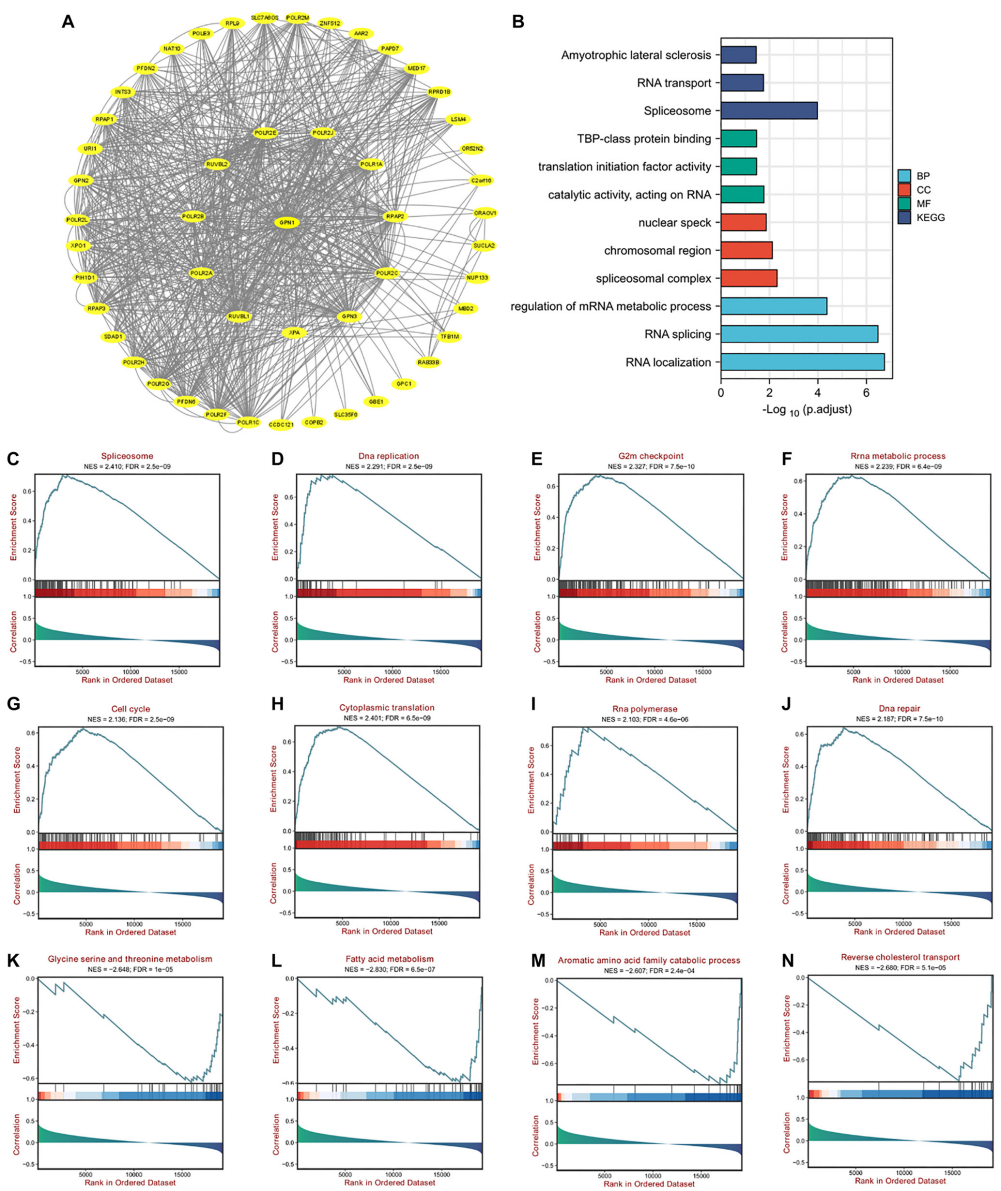
**Functional enrichment analysis and pathways of GPN1 in HCC.** (A) PPI network of GPN1 from the GEPIA dataset and using Cytoscape; (B) GO and KEGG analyses of GPN1; (C–N) GSEA enrichment analysis of GPN1 in HCC. HCC: Hepatocellular carcinoma; PPI: Protein–protein interaction; GSEA: Gene Set Enrichment Analysis; GEPIA: Gene expression profiling interactive Analysis; KEGG: Kyoto Encyclopedia of Genes and Genomes.

Using the GEPIA database, we generated and visualized the PPI network for GPN1 with Cytoscape (Figure 8A). Based on GEPIA data, we identified the top 100 GPN1 co-expressed genes across pan-cancer types and performed GO and KEGG analyses. Results indicated that GPN1 is primarily involved in RNA localization, RNA splicing, the spliceosome, and the regulation of mRNA metabolic processes (Figure 8B). Furthermore, GSEA revealed that GPN1-associated signaling pathways are most enriched in RNA synthesis, cell cycle, RNA and DNA regulation, and metabolism-related processes. These include the spliceosome, DNA replication, rRNA metabolic process, cell cycle, cytoplasmic translation, and glycine-serine and threonine metabolism (Figure 8C–8N).

### Drug sensitivity assessment

**Figure 9.**
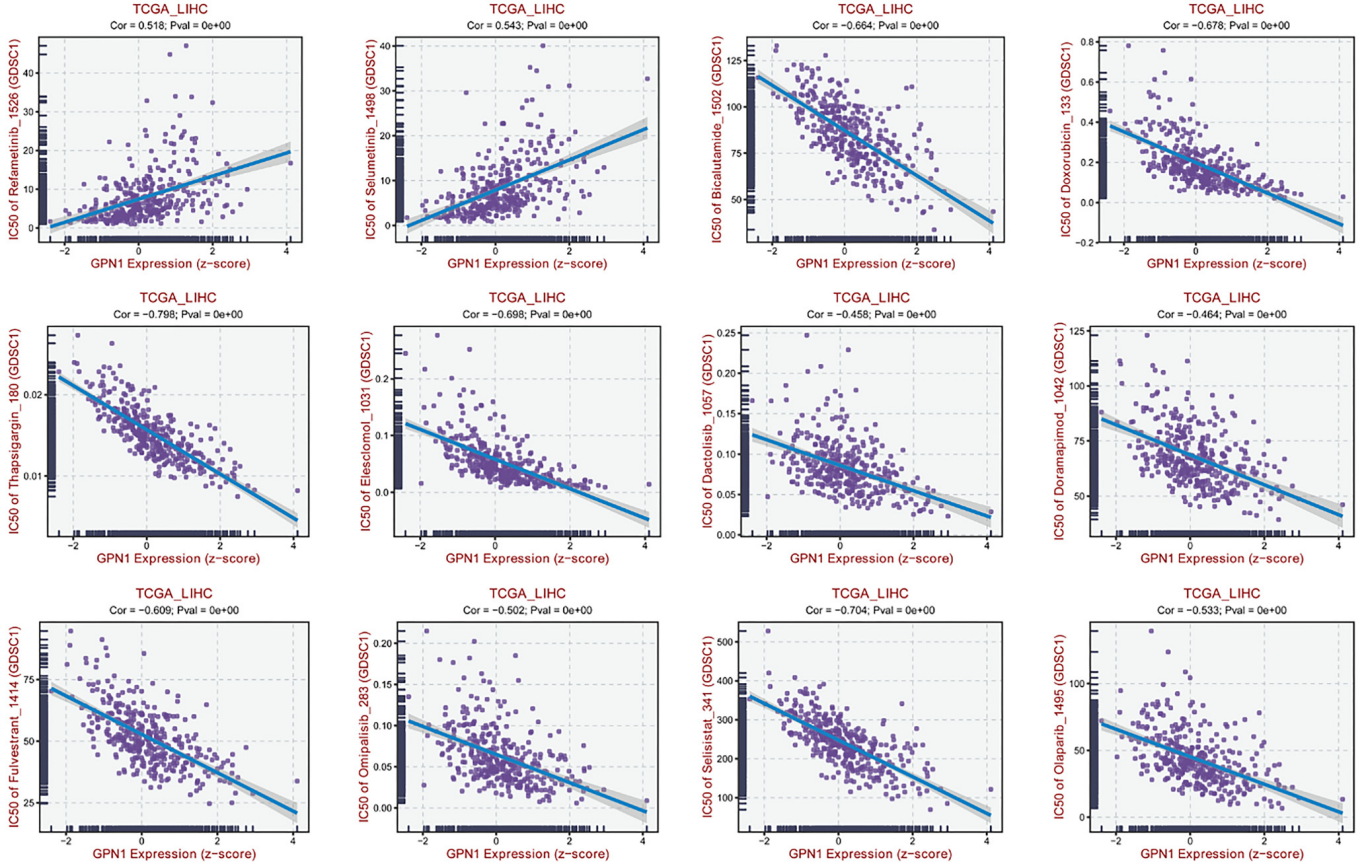
**Drug sensitivity assessment with GPN1 expression.** TCGA: The cancer genome atlas

To investigate whether GPN1 expression is associated with drug sensitivity in HCC, we analyzed the correlation between the risk score and the IC50 values of several potential HCC treatments. The results showed that 12 drugs had a significant correlation with GPN1 expression. Specifically, a positive association was observed between GPN1 expression and the IC50 values of two drugs: refametinib and selumetinib (Figure 9). This suggests that HCC patients with high GPN1 expression may exhibit reduced sensitivity to refametinib and selumetinib, which could be important for guiding clinical drug selection.

### GPN1 expression promotes HCC cell migration

**Figure 10.**
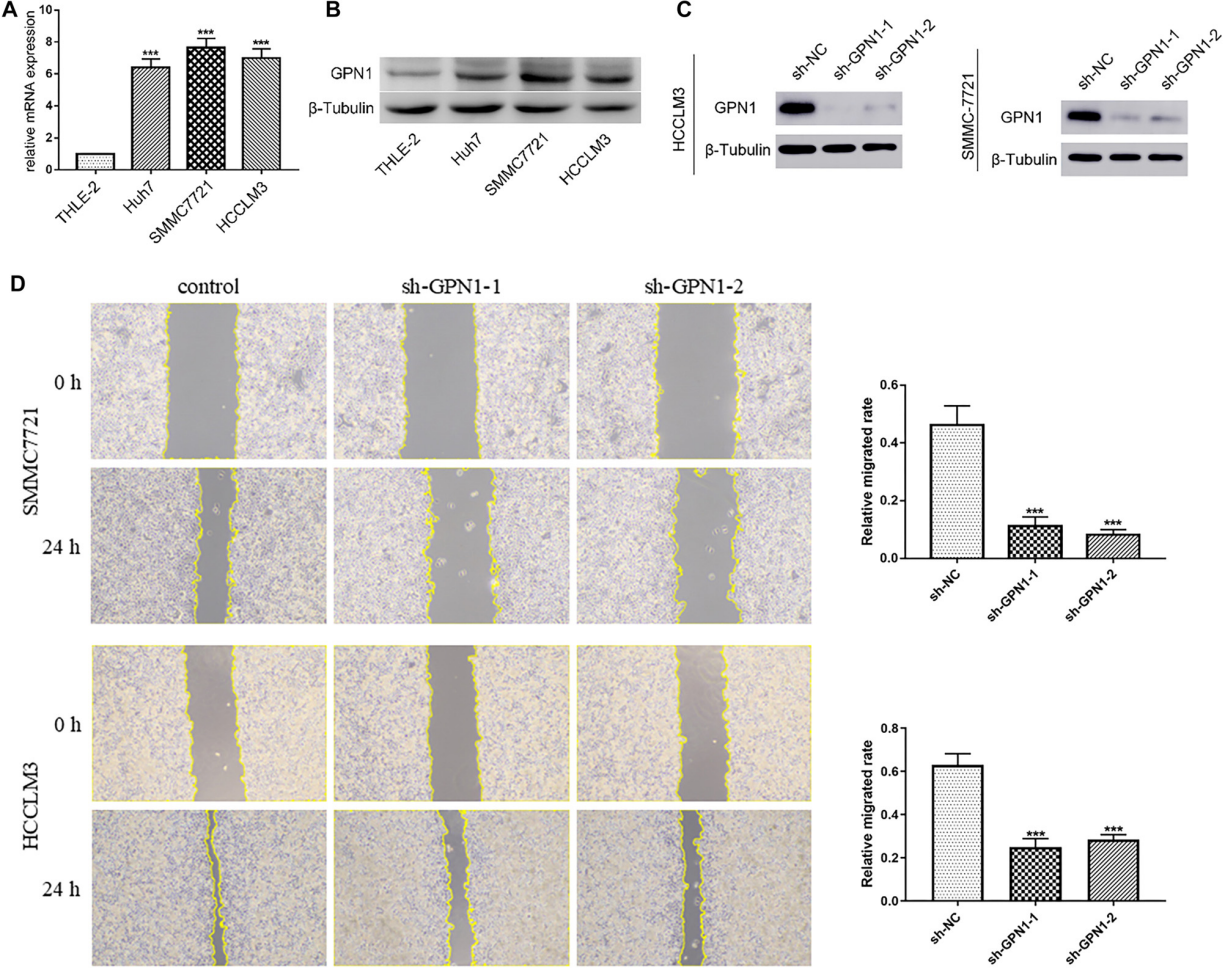
**GPN1 was upregulated in HCC cells and promoted migration in HCC.** (A) qPCR confirmed the high expression of GPN1 in HCC; (B) WB verified the high expression of GPN1; (C) WB analysis showed a decrease in the expression of GPN1 protein in HCC-LM3 cells and SMMC-7721 with GPN1-knockdown; (D) Knockdown of GPN1 inhibited the migration of HCC cells in wound healing assays. WB: Western blotting; HCC: Hepatocellular carcinoma; qPCR: Quantitative PCR

**Figure 11.**
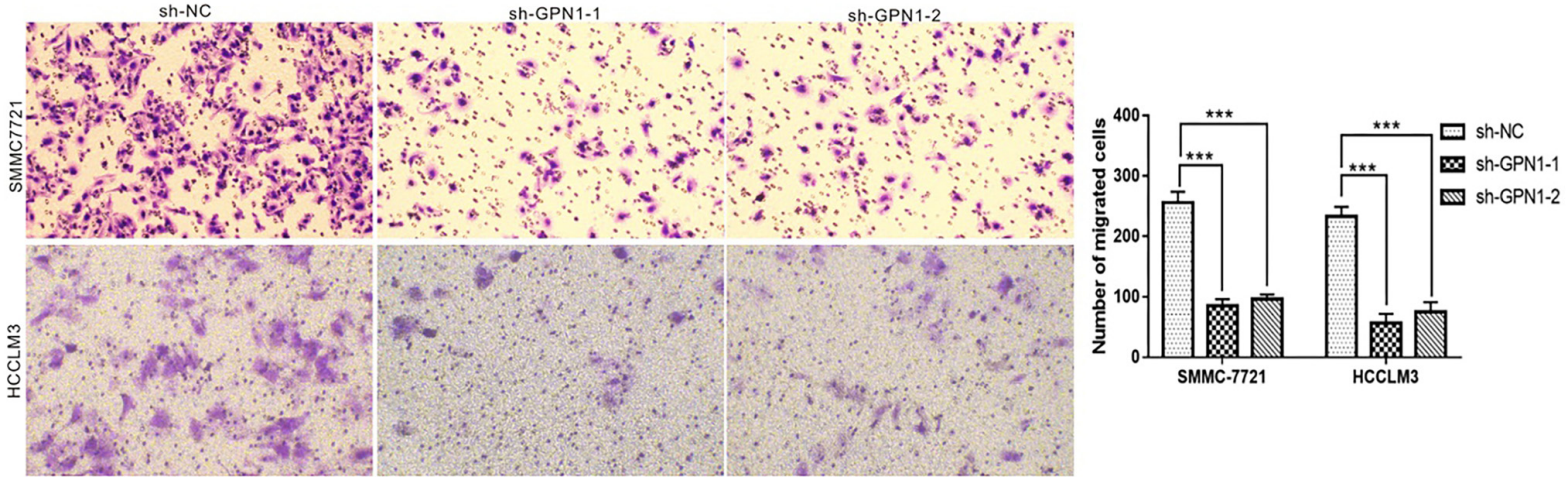
**GPN1 promoted migration of HCC cells. Knockdown of GPN1 inhibited the migration of HCC cells in Transwell assays.** HCC: Hepatocellular carcinoma.

The results of qPCR and western blot analyses demonstrated that GPN1 was highly expressed in Huh-7, SMMC7721, and HCCLM3 HCC cell lines compared to the THLE-2 hepatocyte cell line (Figure 10A and 10B). To investigate the role of GPN1 in HCC, two shRNAs targeting different sites of the GPN1 gene were designed to knock down GPN1 expression in HCC cells. Knockdown efficiency was assessed by western blot, with results indicating that shRNA-GPN1 achieved the highest transfection efficiency (Figure 10C). After GPN1 knockdown, the migration abilities of SMMC7721 and HCCLM3 cells were significantly reduced compared to the control group in wound healing assays (Figure 10D). Additionally, GPN1 knockdown inhibited HCC cell migration in transwell assays (Figure 11). These findings confirm that GPN1 is highly expressed in HCC cells and is associated with increased cancer cell migration.

## Discussion

Our study found that GPN1 expression was significantly elevated in 19 out of 28 cancer tissue samples. Consistent with these findings, data from the TCGA and GEO databases, as well as three HCC cell lines, also demonstrated high levels of GPN1 expression. Across multiple tumor datasets, and especially in HCC, GPN1 gene and protein expression were markedly increased, suggesting that GPN1 could serve as a potential diagnostic biomarker for various cancers.

We also observed a significant association between GPN1 expression levels and OS, pathological grade, pathological stage, and AFP levels. Higher GPN1 expression correlated with worse prognosis and more advanced pathological grades. Cox regression analysis further revealed that GPN1 expression was associated with pathological stage, histological grade, AFP levels, and OS. Kaplan–Meier survival curves indicated that patients with elevated GPN1 expression had significantly lower OS, disease-free survival (DFS), and progression-free intervals. Using these risk factors, we developed a nomogram to predict the 1-year survival probability for HCC patients. The AUC values for predicted survival rates at one, three, and five years were moderate, supporting the potential utility of GPN1 as a prognostic biomarker for HCC.

Based on our bioinformatics analysis, we found that GPN1 expression was associated with clinical characteristics of HCC. Cox regression analysis indicated that GPN1 expression levels in HCC were correlated with pathological T stage, histological grade, and AFP levels. Specifically, higher pathological grades and AFP levels ≥ 400 mg/L were associated with significantly increased GPN1 expression, suggesting a role for GPN1 in promoting HCC cell migration. Supporting these findings, wound healing and transwell assays confirmed GPN1’s involvement in HCC cell migration.

GPN proteins contain five highly conserved G motifs, designated G1 through G5. Each of these GTPases contains a Gly-Pro-Asn (GPN) amino acid insertion at the center of its core fold sequence, which is essential for nucleotide binding and GTP hydrolysis [12]. Altering the GPN gene sequence or modifying the G1 domain of GPN1 disrupts its GTPase activity, leading to the accumulation of RNAPII in the cytoplasm. When active, GPN1 binds RNAPII more effectively, but mutations in the GTPase domain can significantly impair this binding [8, 24].

Our genetic alteration analysis identified changes in the GPN1 gene across different cancers, primarily mutations and amplifications. In HCC, however, almost all alterations were gene amplifications. These findings confirm that GPN1 gene amplification plays an important role in HCC progression.

To perform assembly and stabilization functions, GPNs may operate within a network of molecular chaperones known to interact with RNA polymerases [25]. GPN1, for example, binds to RNAPII in the cytoplasm via GTP binding, which triggers the nuclear import of RNAPII and is driven by GTP hydrolysis. Following this, GPN1 is exported back to the cytoplasm through exportin 1 [10, 26]. Functional enrichment analysis revealed that GPN1 is primarily involved in RNA localization, RNA splicing, the spliceosome, and the regulation of mRNA metabolism. These findings underscore the critical role of GPN1 in mRNA synthesis and the processing of various snRNAs by regulating RNAPII function.

In recent years, significant progress has been made in molecular targeted drugs and systemic immunotherapy for liver cancer [27–29]. However, it remains challenging to base clinical decision making solely on specific biomarkers [30]. The prognosis for HCC is still poor, making it the third leading cause of cancer-related deaths worldwide [2]. Cancer progression and clinical outcomes are heavily influenced by the tumor microenvironment, which consists not only of tumor cells but also various non-tumor cells, including tumor-infiltrating immune cells that regulate inflammation and cancer progression [31–33]. The immune system plays a key role in modulating HCC progression [30]. Our results indicate that GPN1 overexpression in HCC significantly affects immune infiltration. Specifically, GPN1 expression in HCC was associated with cytotoxic cells, DCs, Th2 cells, CD8+ T cells, CD4+ T cells, and neutrophils. Both CTLA-4 and PD-1 are critical in tumor immunotherapy [34]. Additionally, mutations in the TP53 gene are common in cancer, where they suppress antitumor immunity and reduce the effectiveness of cancer immunotherapy [35, 36]. Our study found an association between TP53 mutations and GPN1 expression in HCC, and heat map analysis showed a positive correlation between TP53 and GPN1 expression in immune checkpoints. Therefore, GPN1 overexpression may influence immunotherapy outcomes for HCC. We confirmed that GPN1 is associated with responses to anti-PD-1/CTLA-4 and anti-PD-L1 therapies.

Drug sensitivity assessments further showed a significant correlation between GPN1 expression and the efficacy of 12 drugs, including molecular-targeted therapies and chemotherapies. Among these, the IC50 values of refametinib and selumetinib were positively correlated with GPN1 expression, suggesting that high GPN1 expression in HCC patients may indicate a poorer response to these drugs. Overall, these results suggest that GPN1 is involved in tumor immune infiltration and could serve as a potential biomarker to guide molecular targeted therapy and systemic immunotherapy in HCC.

To the best of our knowledge, this study is the first to explore the potential biological function of GPN1 in HCC through both bioinformatics analysis and *in vivo* experiments. However, some limitations should be acknowledged. GPN1’s role as a regulator of RNAP II nuclear import is critical, meaning that its suppression may lead to a wide range of functional consequences beyond just affecting cell migration. Therefore, a series of multifaceted experiments should be conducted in the future to examine the broader effects of GPN1 knockdown on HCC cells. Additionally, the downstream target molecules and signaling pathways of GPN1 in HCC remain unclear. Further *in vivo* and *in vitro* studies are needed to clarify the molecular mechanisms by which GPN1 contributes to HCC progression.

## Conclusion

The results of this study suggest that GPN1 could serve as a valuable pan-cancer diagnostic and prognostic biomarker, based on its correlation with clinicopathological characteristics, tumor immune cell infiltration, and findings from functional enrichment analyses. Notably, this study is the first to document the upregulation of GPN1 in HCC cells. We also confirmed that GPN1 expression promotes HCC cell migration. Additionally, GPN1 expression levels were associated with tumor infiltration status and response to immunotherapy. Taken together, these data indicate that GPN1 may serve as a robust diagnostic and prognostic biomarker and a potential therapeutic target in HCC.

## Data Availability

Publicly available datasets were analyzed in this study. The original contributions presented in the study are included in the article. Further inquiries can be directed to the corresponding authors.
